# Bone-kidney axis: A potential therapeutic target for diabetic nephropathy

**DOI:** 10.3389/fendo.2022.996776

**Published:** 2022-10-24

**Authors:** Ming Yang, Shilu Luo, Jinfei Yang, Wei Chen, Liyu He, Di Liu, Li Zhao, Xi Wang

**Affiliations:** ^1^ Department of Nutrition, Xiangya Hospital, Central South University, Changsha, China; ^2^ Department of Nephrology, The Second Xiangya Hospital of Central South University, Changsha, China; ^3^ Department of Reproduction and Genetics, The First Affiliated Hospital of Kunming Medical University, Kunming, China; ^4^ National Clinical Research Center for Geriatric Disorders, Xiangya Hospital, Central South University, Changsha, China

**Keywords:** bone, diabetic nephropathy, bone-derived hormone, FGF23, lipocalin 2

## Abstract

Diabetic nephropathy (DN) is the leading cause of end-stage renal disease (ESRD). However, its pathogenesis remains unclear, and effective prevention and treatment strategies are lacking. Recently, organ-to-organ communication has become a new focus of studies on pathogenesis. Various organs or tissues (the liver, muscle and adipose tissue) secrete a series of proteins or peptides to regulate the homeostasis of distal organs in an endocrine manner. Bone, an important part of the body, can also secrete bone-derived proteins or peptides that act on distal organs. As an organ with high metabolism, the kidney is responsible for signal and material exchange with other organs at any time through circulation. In this review, we briefly discussed bone composition and changes in bone structure and function in DN and summarized the current status of bone-derived proteins and their role in the progression of DN. We speculated that the “bone-kidney axis” is a potential target for early diagnosis and treatment of DN.

## Introduction

Diabetic nephropathy (DN) is characterized by serious renal microangiopathy caused by diabetes (1–3). With the increasing incidence of diabetes worldwide, the number of patients with DN is also gradually increasing. Moreover, DN is the leading causes of end-stage renal disease (ESRD) and imposes a heavy economic burden on society (4). At present, the treatment of DN lacks of specific drugs, especially symptomatic treatment. Therefore, it is necessary to understand the pathogenesis of DN and develop effective strategies for early diagnosis and prevention.

To date, studies focused on the kidney as an independent organ to explore the molecular mechanisms underlying renal pathological changes in diabetes mellitus (5). However, the human body is an organic whole, and signal and material exchange between the kidney and other organs occurs through blood circulation. As an important part of the human body, bone plays a locomotive, supportive and protective role. Recent studies have revealed that bone can secrete various bone-derived polypeptides and proteins to regulate of other organs in an endocrine manner (6–8). In the advanced stage of kidney disease, owing to the disorder of calcium and phosphorus metabolism and abnormal hormones, bones undergo corresponding secondary pathological changes, known as chronic kidney disease mineral bone disease (CKD-MBD) (9–11). However, the role of bone as an endocrine organ in DN, especially in the early stage, remains elusive. In this review, we summarized some important bone-derived proteins and discussed their role in DN, thus providing new therapeutic targets for the treatment of DN.

## Brief description of bone tissue

Bones are the strong organs of vertebrates and play an important role in the locomotion, support and protection of the body. Bone consists of two main parts as follows: the extracellular matrix composed of organic and inorganic components and cellular components (12–14). Collagen I fibers, non-collagen glycoproteins and proteoglycan constitute the organic portion of bone, whereas calcium and phosphate (Pi) carbonates constitute the inorganic portion (15). In addition, bone is composed of three types of cells, namely, osteoclasts, osteoblasts and osteocytes (16–18). In the body, bone structure is not static but in a dynamic process of decomposition and reconstruction. If this balance is disrupted, such as when the rate of bone resorption is higher than the rate of bone formation, bone mass decreases and bone fragility increases, which may lead to osteoporosis and fractures (19, 20). Bone cells are involved in the formation and maintenance of bone structure. Osteoclasts are polarized multinucleated giant cells that are primarily involved in the absorption of the bone matrix (21, 22). Bone resorption mainly involves the adhesion of osteoclasts to the bone matrix, acidification of bone resorption lacunae and depredation of organic components (15). In addition to their role in bone resorption, osteoclasts are involved in regulating the differentiation of osteoblasts and mobilization of haematopoietic progenitors (23, 24). Osteoblasts are the main functional cells for bone formation and are responsible for the synthesis, secretion and mineralization of the bone matrix. They are derived from pluripotent mesenchymal stem cells (MSCs). Differentiation of osteoblasts is regulated by multiple signaling pathways, and their content does not exceed 6% of the total number of bone cells (15, 25). After the resorption of osteoclasts to form bone lacunae, osteoblasts migrate to the resorption site and secrete bone matrix, which mineralizes to form new bones (26, 27). Osteocytes are the most abundant cells in bone, accounting for approximately 90% of bone cells (28, 29). They have a unique protuberant structure, which helps them to form interconnected tubule networks and communicate with each other through intercellular interstitial junctions (30). Osteocytes act as receptors that convert external mechanical stimuli to biochemical signals that are transmitted to other effectors through a network of tubules (31, 32). In fact, they can regulate bone formation and resorption by influencing the function of osteoclasts and osteoblasts (25).

**Figure 1:**
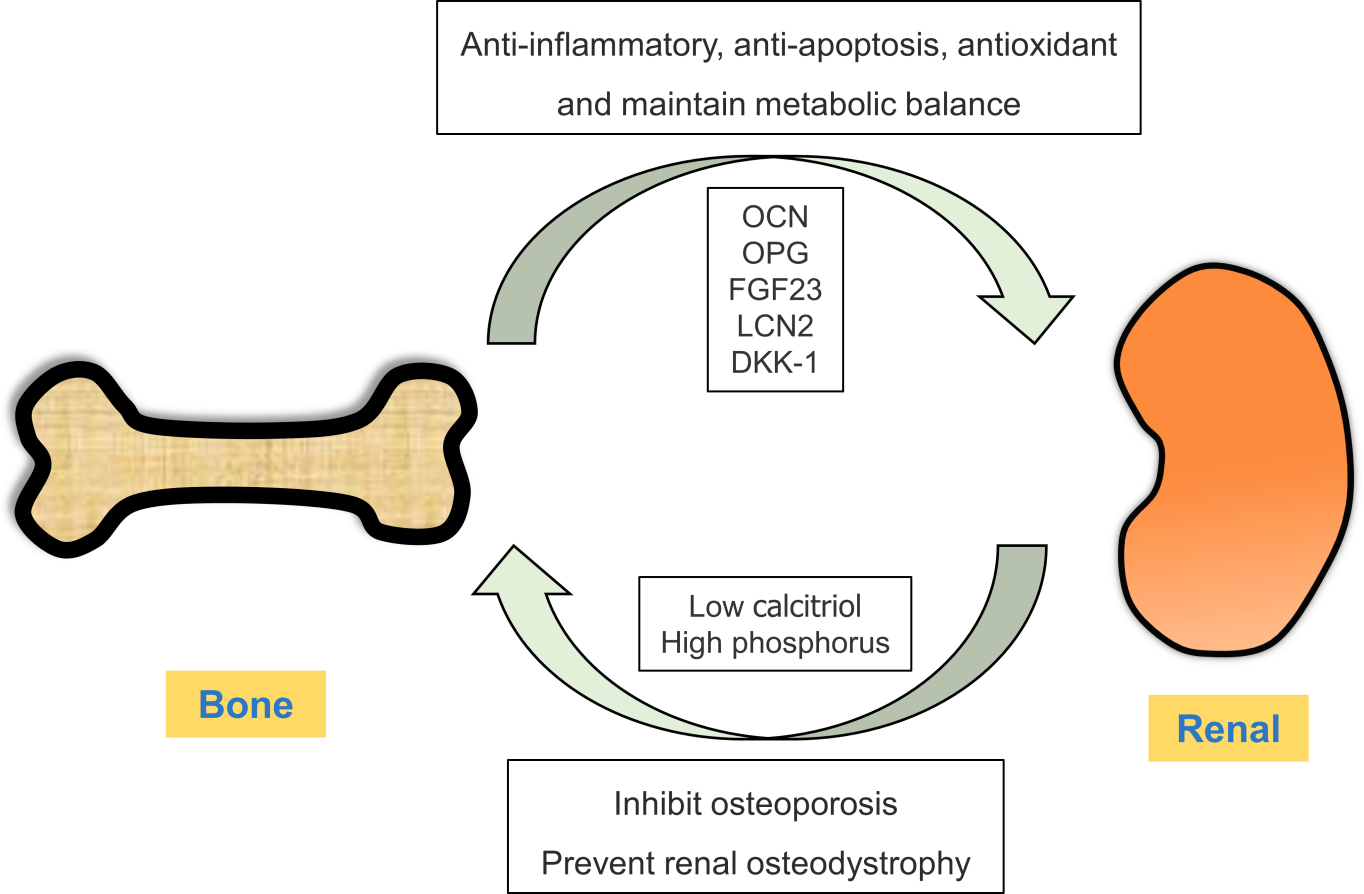
Crosstalk between the bone and kidney. Under physiological conditions, bones can secrete bone-derived peptides or proteins (OCN, OPG, FGF23 and LCN2) into the kidney through circulation. In ESRD, renal insufficiency results in low calcitriol and high phosphorus levels, thereby disrupting the balance between bone synthesis and resorption.

Bone mainly plays a role in supporting and protecting the viscera and regulating calcium and phosphorus metabolism (33, 34). However, recent studies have shown that bone not only acts as the target organ of several endocrine glands or tissues through hormone regulation, but also produces and secretes a large number of bioactive substances, such as bone regulatory proteins, active peptides, growth factors and hormones (35). These substances can regulate bone homeostasis in both autocrine and paracrine manners and act on the distal target organs in an endocrine manner, thus playing a role in regulating their biological activity (36). Cellular active substances secreted by bone as an endocrine organ and their relationship with kidney diseases are described below (**Figure 1**).

## DN and bone metabolism

As a systemic metabolic disease, diabetes mellitus has adverse effects on bone metabolism and remodeling. Patients with diabetes have an increased risk of fractures, reduced osteoblast recruitment and increased osteoclast production (22213724). In addition, drugs used in the treatment of diabetes can affect the bone; in particular, thiazolidinediones (TZDs) can enhance the differentiation of mesenchymal cells into adipocytes instead of osteoblasts, thus increasing the risk of osteoporosis (37, 38). The kidney is an important organ regulating bone homeostasis, and abnormal bone metabolism owing to kidney diseases results in “renal osteodystrophy”. Renal osteodystrophy is defined as a series of skeletal disorders caused or aggravated by CKD, resulting in bone fragility and fractures, abnormal mineral metabolism, and exoskeletal manifestations (39, 40). Mechanistically, a decline in renal function leads to phosphate retention in patients with CKD, and the production of 1, 25 dihydroxy vitamin D3 (calcitriol) is directly inhibited owing to high phosphorus levels and renal insufficiency. These adverse changes lead to a decrease in calcium levels and secondary hyperparathyroidism, eventually leading to abnormal bone metabolism (41). Therefore, the structure and function of bone tissue are also altered in the diabetic kidney, which may lead to abnormal secretion of bone-derived hormones.

## Bone-derived hormones and DN

### Osteoprotegerin

Osteoprotegerin (OPG) is a cytokine of the tumor necrosis factor (TNF) receptor superfamily, which is also known as osteoclastogenesis inhibitory factor (OCIF) (42, 43) or TNF receptor superfamily member 11b (TNFRS11B) (44, 45). Structurally, OPG is a 60-kDa secreted glycoprotein composed of 401 amino acids and can be assembled on the cys-400 residue of the heparin-binding domain to form a 120-kDd disulfide linked dimer for secretion (46). Before it is secreted, the 21-Aa signal peptide at the N-terminal is cleaved to form a 380-Aa mature OPG protein (47, 48). Therefore, three forms of OPG are involved in the cycle: 60-kDa monomer, 120-kDa disulfide bound to a homodimer and OPG bound to receptor activator of nuclear factor-κB ligand (RANKL) and TRAIL (ligands of OPG) (49, 50). Functionally, OPG can inhibit the activation of osteoclasts, thereby inhibiting the formation and differentiation of osteoclast and bone resorption (51). In addition, it is involved in the development of cardiovascular diseases (52, 53). The biological function of OPG is mediated mainly through the OPG/RANKL/RANK signaling pathway. RANKL is a type II homologous trimer transmembrane protein, which is mainly expressed as a membrane binding and secretory protein (54). RANK is a type I homologous trimer transmembrane protein that is highly expressed on mature osteoclasts (55). Under normal conditions, RANKL binds to RANK on the surface of osteoclasts and subsequently recruits TRAF6 (the connector protein), resulting in the activation and translocation of NF-κB to the nucleus, which eventually triggers the transcription of osteoclast-related genes (56). OPG can competitively bind to RANK, thus blocking the effects of RANKL (57).

In addition to inhibiting osteoclasts, OPG is closely related to the occurrence and progression of kidney diseases. Chang et al. reported that serum OPG levels were significantly higher in DN patients with microalbuminuria and macroalbuminuria than in those with normal albuminuria (58). In addition, a clinical study revealed that the all-cause mortality rate was significantly higher among DN patients with high OPG levels than among those with low OPG levels, and GFR was higher in patients with high levels of plasma OPG (59). Similar results were observed in a study in which immunohistochemical (IHC) staining of renal biopsy tissue of DN patients revealed that OPG was mainly expressed in renal tubules instead of the glomerulus, and the expression of OPG was higher in patients with albuminuria than in those with microalbuminuria (60). Therefore, OPG may play an adverse role in the progression of DN. Furthermore, studies employing oligonucleotide arrays have showed that OPG expression is significantly different between patients with DN and control group (61). In an *in vivo* study, the expression of pro-inflammatory (IL-6) and pro-fibrotic mediators (TGF-β) in the kidney was higher in OPG-treated mice than in control mice, which suggests a relationship between OPG and the progression of DN. However, a few studies have investigated the molecular mechanisms of OPG underlying the progression of DN. Therefore, the following question should be addressed in future studies: How does the high expression of OPG in the kidney activate the downstream pathway to promote or inhibit the progression of DN?

### Osteocalcin

Osteocalcin (OCN), also known as bone gamma-carboxyglutamic acid (Gla), is a circulating protein secreted by osteoblasts (62, 63). It is initially synthesized with 95 amino acids and is subsequently cleaved to form a 46-amino acid mature peptide containing three gamma-carboxyl glutamate residues at sites 13, 17 and 20 (64, 65). In humans, mature OCN peptides have 49 amino acids and can be gamma-carboxylated at sites 17, 21 and 24 (66). The degree of gamma-carboxylation increases the binding ability of OCN to the mineral components of the extracellular matrix, resulting in the accumulation of gamma-carboxylated OCN in bones (67). An acidic microenvironment is created during osteoclast-driven bone resorption. Under this condition, OCN is decarboxylated, and its affinity for mineral components of the extracellular matrix is reduced. As a result, OCN is released into circulation as an endocrine hormone that regulates the function of distal target organs (68, 69). OCN was initially thought to play a role in mineralisation of the extracellular matrix and was considered a serum marker of osteoblast-driven bone formation (70). Ducy et al. demonstrated that the rate of bone formation increased in osteocalcin-deficient mice without compromising bone absorption (71). However, recent studies have shown that OCN acts as a hormone through an endocrine pathway.

The role of OCN as an endocrine hormone was first demonstrated by Lee et al., who reported increased blood glucose levels, fat mass, glucose intolerance and insulin resistance in osteocalcin-deficient mice compared with control mice. However, the loss of the protein tyrosine phosphatase (OST-PTP) in Esp-/- mice resulted in the opposite phenotype characterized by: decreased blood glucose levels, reduced fat mass and improved islet beta cell function, thereby reducing the effects of obesity and glucose intolerance (72). The reason for this contradictory reverse phenotype is that the encoded phosphatase is a negative regulator of OCN activation (72). Therefore, OCN is critical for maintaining insulin secretion and glucose homeostasis. Consistently, exogenous OCN can also maintain metabolic homeostasis. Wei et al. reported that exogenous OCN treatment increased the expression of the insulin 2 (Ins2) gene and insulin secretion in islet cells, these OCN-induced changes were blocked in Gprc6a-deficient islets. Mechanistically, OCN can regulate β-cell replication in a cyclin D1-dependent manner through the Gprc6a receptor in islet cells (73). Similar results were observed in a study in which long-term treatment of OCN significantly alleviated the harmful effects of high-glucose and high-fat diets on body weight and glucose metabolism in mice (74). In addition to its effects on glucose metabolism, abnormal expression of OCN is associated with reproductive function. Oury et al. reported that the size of litters and the number of pups per litter were smaller when male OCN^−/−^129Sv mice were bred with wild-type female mice. In addition, the testes were smaller, the sperm count was lower and the expression of testosterone biosynthesis genes was significantly lower in male OCN^−/−^ mice than in male of wild-type mice (72). Muscle mass was reduced in OCN^−/−^ mice compared with wild-type mice, with a decrease in the average area of muscle fibers (75).

Because DN is a metabolic disorder, changes in plasma OCN concentration are also closely related to its progression. Inukai et al. reported that OCN levels were elevated in patients with early-stage DN without serum creatinine elevation (76). Another study demonstrated that eGFR is negatively correlated with OCN concentration in patients with diabetes (77). Therefore, given that serum OCN levels are increased in DN, OCN may serve as a biomarker for predicting the progression of DN. On the other hand, OCN concentration may increase owing to an increase in compensatory OCN secretion caused by high blood glucose levels; On the other hand, OCN excretion is reduced and it is retained in the body owing to a decline of renal function during the progression of DN. Although a few studies have investigated the molecular mechanisms of OCN in DN, it plays an important role in the kidney, especially in DN, as an endocrine factor of bone origin.

### Fibroblast growth factor 23

Fibroblast growth factors (FGFs) play diverse roles in a wide range of biological processes by activating FGF receptor tyrosine kinases (FGFRs) (78). FGF23, a member of the FGF family, is mainly produced by osteocytes and osteoblasts in bone and participates in the regulation of phosphate homeostasis (79). In humans, the FGF23 gene consists of three exons and a 10-kb genome sequence encoding a precursor protein of 251 amino acids that is secreted from bone into circulation (80). FGF23 is a 32-kDa glycoprotein that contains a proteolytic site and the proteolytic cleavage of FGF23 is regulated by O-glycosylation and phosphorylation (78). The cleavage of FGF23 is inhibited by O-glycosylation induced by N-acetylgalactosaminyltransferase 3 (GALNT3), which eventually increases the levels of full-length FGF23 protein in circulation (81). *In vivo* studies have shown that FGF23 can be cleaved to produce a C-terminal and an N-terminal fragment. The full-length FGF23 protein is biologically active, whereas the cleaved fragment is inactive; however, the C-terminal fragment retains its ability to bind to FGF23 receptors (82). The kidney is the main target organ of FGF23. Circulating FGF23 binds to the FGFR1/Klotho complex in renal cells to regulate phosphorus homeostasis. The binding of FGF23 to the FGFR1/Klotho complex activates various kinases, such as serum glucocorticoid-regulated kinase-1 (SGK1), and down-regulates the type II sodium-dependent phosphate (NaPi) cotransporters NaPi 2a and NaPi 2c in proximal tubules, thereby inhibiting the reabsorption of phosphate (Pi) (83, 84). In addition to increasing Pi excretion through urine, FGF23 can inhibit 1α-hydroxylase, thereby reducing the production of 1,25(OH)2D and indirectly inhibiting Pi absorption in the intestines (84). Furthermore, FGF23 can reduce the secretion and synthesis of parathyroid hormone (PTH), which in turn directly increases FGF23 levels by acting on osteoblasts/osteocytes. Therefore, the three hormones in the body that control calcium and phosphorus metabolism (PTH; 1,25(OH)2D and FGF23) interact to form a feedback loop among the kidney, bone, gut, and parathyroid glands (85–87). FGF23 levels ranging from 50 to 200 pg/mL are required to maintain normal mineral homeostasis. Elevated FGF23 levels can cause hypophosphatemia and rickets and decrease the levels of 1,25(OH)2D, whereas excessively low levels of FGF23 can hyperphosphatemia and soft tissue calcification and increase the levels of 1,25(OH)2D (88).

The serum concentration of FGF23 is altered in DN patients with kidney injury. Inci et al. reported that serum FGF23 levels were significantly higher in patients with type 2 diabetes compared with controls and were associated with urinary albumin levels (89). In addition, FGF23 concentrations is also associated with an increased risk of cardiovascular diseases and death in patients with DN (90). Tsai et al. reported that high FGF23 levels were associated with low hemoglobin levels in patients with stage 3 and 4 CKD (91). The mechanism underlying the increase in FGF23 levels has been partially revealed. Disorder of phosphorous metabolism can result in a secondary increase in FGF23 levels. Mace et al. showed that plasma FGF23 levels increased 2.5 times within 15 minutes of removal of both kidneys. In addition, the removal of one kidney led to an increase in blood FGF23 levels compared with control rats (92). These studies suggest that normal renal function is critical for maintaining FGF23 concentration. In a similar study reporting on the relationship between renal insufficiency and increased FGF23 concentration, nephritic rats were injected with recombinant FGF23, a prolonged disappearance curve was observed and the half-life of FGF23 increased from 4 minutes to 12 minutes (92). These results indicate that impaired renal function reduces the excretion rate of FGF23 through the kidney and hence increases the serum concentration of FGF23. In addition to the decreased rate of renal excretion, other factors can influence FGF23 concentration. Sørensen et al. reported that circulating FGF-23 levels were elevated in patients with type 2 diabetes with normal or mildly impaired renal function, which is associated with impaired diastolic function and decreased myocardial blood flow (93). Moreover, in earl-stage DN, increased serum FGF23 levels, instead of urinary albumin, may serve as more accurate biomarker for predicting the progression of the disease (94).

In addition to regulating mineral metabolism, FGF23 is involved in various of cellular processes. In a study, renal damage and fibrosis were alleviated and the levels of inflammatory cytokines in serum and renal tissue were significantly lower in DB/DB mice (a mouse model of DN) injected with exogenous C-terminus of FGF23 (compared with control mice) (95). As mentioned earlier, because the C-terminus of FGF23 has no biological activity, it competes with intact FGF23 for binding to FGF23 receptors, which suggests that increased FGF23 levels in DN may aggravate kidney damage by promoting the activation of inflammation. Recent studies have highlighted the important role of FGF23 as a bone-derived factor in the progression of DN; however, its molecular mechanism in addition to regulating calcium and phosphorus metabolism warrants further investigation.

### Lipocalin 2

In addition to regulating mineral metabolism, bone plays a key role in maintaining energy and glucose metabolism by secreting of bone-derived hormones. Lipocalin 2 (LCN2) is a hormone that is mainly expressed on osteoblasts, and its level is > 10 times higher in osteoblasts than in other tissues in the basal state. Osteoblast-specific knockout of LCN2 in mice can increase food intake, fat mass and body weight, which is accompanied by glucose intolerance, insulin resistance and pancreatic β cell dysfunction, eventually resulting in decreased insulin secretion after glucose stimulation (96, 97). LCN2, also known as siderocalin, neutrophil gelatinase-associated lipoprotein (NGAL) or uterine calcitonin, is a glycoprotein composed of 198 amino acids. It is encoded by a gene located at chromosome 9 locus 9q34.11, which produces numerous functional transcripts (98). LCN2 exists in many molecular forms, including mono-dimer, dimer or heterodimer, which can bind to neutrophil gelatinase B to form disulfide bonds (99). In the kidney, LCN2 is considered an important biomarker for predicting the progression of DN. Najafi et al. reported that serum NGAL levels were significantly higher in the DN patients with macroalbuminuria than in those with normoalbuminuria and microalbuminuria (100). In addition, urinary NGAL (LCN2) levels were higher in patients with type 2 diabetes than in those without diabetes and higher in patients with urinary albumin than in those without urinary albumin (101). However, the molecular mechanism and role of elevated NGAL levels in DN warrant further investigation. Studies have shown that damaged kidney cells in kidney diseases may secrete high amounts of LCN2 (102, 103), and neutrophils and macrophages may also be the source of lCN2 elevation (104). However, the role of bone-derived LCN2 (the major producer of resting LCN2) in renal diseases remains unclear. Moreover, the increase in LCN2 levels in DN may be a compensatory protective effect. Liu et al. demonstrated that in high-concentration glucose stimulation in NGAL-knockout HK-2 cells significantly increased oxidative stress and the secretion of interleukin-6 (IL-6), fibronectin (FN) and type IV collagen (105). In addition, compared with wild-type mice with diabetes, diabetic NGAL^-/-^ mice had deteriorated renal function, more severe glomerulosclerosis and tubular vascular degeneration (105). These results suggest that LCN2 plays an indispensable role in the progression of DN. However, the role of bone-derived LCN2 in kidney diseases warrants further investigation (**Table 1**).

**Table 1:** Some bone-derived hormones as currently defined.

Bone-derived proteins	References
Osteoprotegerin	(106)
Osteocalcin	(107)
osteopontin	(108)
FGF23	(109)
Lipocalin 2	(110)
Sclerostin	(65)
Dickkopf-1	(111)
Bone morphogenetic protein 2	(112)

## Conclusion

In recent years, the crosstalk between organs has gradually become a new focus of research into the pathogenesis of diseases. In addition to the traditional endocrine organs, other organs such as bone and muscle can secrete relevant peptides or proteins in an endocrine manner to participate in the maintenance of homeostasis in the human body. In addition to locomotion and support, bone can secrete bone-derived factors to regulate the metabolism of the body. Bone metabolism is abnormal and the endocrine function of bones is impaired in DN, especially in ESRD. However, only a few studies have examined the role of bone-derived factors in the progression of kidney diseases. Although the role of several bone-derived factors in regulating renal communication has been preliminarily revealed, most studies have only described the phenotypic relationship between the liver and kidney and have not revealed the mechanisms underlying the direct communication between them. In this review, we summarized some important bone-derived proteins and discussed their role in DN and discussed the relationship between bone and kidney diseases. However, several concerns remain unaddressed; for example, how many types of osteogenic factors are there? More precise biological techniques are required to answer such questions. Although it has been reported that the serum concentration of some bone-derived factors is altered in DN, their role in the progression of DN and the underlying molecular mechanisms remain unknown. In conclusion, bone-derived factors offer novel strategies for the diagnosis and treatment of DN, and the “bone-kidney axis” may be used as a therapeutic target for DN in the future.

## Author contributions

MY the first draft of the manuscript. SL, JY, WC, LH, DL, LZ provided consultations on the preparation of the work. XW contributed to manuscript revision, read, and approved the submitted version.

## Funding

This work was supported by the National Natural Science Foundation of China (81900069, 82000697).

## Acknowledgments

We thank Bullet Edits Limited for the linguistic editing and proofreading of the manuscript.

## Conflict of interest

The authors declare that the research was conducted in the absence of any commercial or financial relationships that could be construed as a potential conflict of interest.

## Publisher’s note

All claims expressed in this article are solely those of the authors and do not necessarily represent those of their affiliated organizations, or those of the publisher, the editors and the reviewers. Any product that may be evaluated in this article, or claim that may be made by its manufacturer, is not guaranteed or endorsed by the publisher.
